# Patient knowledge, attitude and behavior towards dental treatment in India

**DOI:** 10.6026/973206300222764

**Published:** 2026-05-31

**Authors:** Pallavi Priya, Imran Md, Vaibhava Raaj, Subhash Kumar, Qalbi Fatima, Abhishek Sinha

**Affiliations:** 1 Department of Dentistry, Patna Medical College & Hospital, Patna, Bihar, India

**Keywords:** Knowledge, attitude and behavior (KAB), dental treatment, patient satisfaction, government hospital, dental outpatient

## Abstract

Limited patient knowledge and inconsistent treatment-seeking behavior remain major challenges in effective dental care delivery in government healthcare settings. Therefore, it is of interest to assess patient knowledge, attitude and behavior toward dental treatment in an outpatient department of a government medical college hospital. Hence, a cross-sectional survey of 450 patients was conducted using a validated questionnaire covering socio-demographic factors and treatment-related perceptions and practices. Only 34.2% demonstrated adequate knowledge, 52.4% showed positive attitudes, while treatment-seeking was predominantly pain-driven with poor follow-up compliance. Thus, we show the need for structured patient education and improved service strategies to enhance treatment adherence and oral health outcomes, which highlights the necessity of organized patient education and service delivery changes at the governmental dental clinics.

## Background:

Access to quality oral healthcare is an essential part of overall health service provision, but there are still high disparities in dental care access, affordability and use across socioeconomic lines, geographic areas and healthcare systems in all countries of the world [1]. In India, where the public-private gap in healthcare provision is strong, government hospitals and medical colleges in India are the main -and in many cases the only - provider of low-cost dental services to the economically disadvantaged sectors of the population, offering a broad spectrum of treatment services, including basic restorative services to complex oral surgery at little or no cost to the patient [2]. Knowledge about the perception, interaction and reaction of the patients to the dental care provided in these government hospitals is important to maximise the service delivery process, enhance patient outcome and overcome the obstacles that stop proper use of available services. Knowledge, Attitude and Behavior (KAB) is a well-known conceptual triad that can be used to develop a solid theoretical framework of factors that determine healthcare utilization and treatment outcomes. Knowledge is the factual information that patients bear about the existing dental procedures, the signs of these procedures, their advantages, constraints and post-surgical needs. Attitude includes the beliefs, perception, feelings and predispositions that patients have about dental treatment, dental professionals and the health care environment. Behavior is the action taken by the patients i.e., their ways of seeking treatment, how they follow the prescribed treatment plans, how they adhere to the instructions given by the post-surgery and how they attend follow-up classes [3]. These three constructs are not always linear in their relationship, which means that an adequate knowledge does not always correspond to the positive attitudes and positive attitudes do not always result in the positive behaviors [4]. Dental treatment literacy on the part of patients in an Indian setting has been reported to be generally poor and most of them have misconceptions on nature, functions and effects of the most common dental procedures. Research in the different states of India has recorded that a great percentage of the population has inaccurate beliefs regarding tooth extraction as the default treatment procedure with regard to dental discomfort, believes that dental procedures are inherently dangerous or harmful, is not aware of other forms of treatment such as restorative treatment and preventive treatment and has little knowledge on the significance of completing and adhering to treatment [5]. These knowledge gaps are enhanced in less educated population and with lesser exposure to health education and the latter is a typical attribute of patients who seek healthcare in government hospitals. The attitudes held by patients towards dental treatment can be described as the result of a constellation of issues such as past experiences in dental care, cultural beliefs, perceived necessity of care, trust of the dental practitioners, fear and anxiety of the process of dental care and perceived quality of the treatment in the facility [6]. DentoPhobia of the dentist, especially dental anxiety has been cited as a major impediment to the use of dental care among groups of people worldwide and the prevalence rates have been reported of between 10 percent and 65 percent depending on the measure tool used and the target population [1]. Other attitudinal determinants in the government hospital environment like perceived waiting duration, quality of communication between patients and healthcare providers and views on the sufficiency of infrastructure facilities might also contribute to patient engagement in dental services [8]. The behavioral aspect of dental health includes the decision to obtain treatment as well as the behavioral pattern of seeking treatment, attendance at treatment course, adherence to overall treatment course, compliance with prescribed medical treatment and post-operative guidelines, follow-up visits and adherence to prescribed medication and oral healthcare measures after the dental care [9]. The gap between initiation and continuation of the treatment is the acute issue of the dental public health since an incomplete treatment, including the unfinished root canal treatment, the partial prosthodontic reconstruction, or an unfinished orthodontic therapy can lead to a worse outcome than no treatment [10]. The state of Bihar, which is a unique socio-demographic area with a high population, low per capita income, high rural population ratio and a small dental workforce in comparison with the population size offers unique challenges to the dental healthcare delivery [11]. Government Medical College and Hospital, Patna, is a large referral center of dentalcare in the state, which accepts patients of various socio-economic status, education and geographical backgrounds in the state of Bihar and beyond. KAB profile of the patients visiting this facility should be realized to detect particular gaps in patient education, consider attitudinal barriers and develop interventions aimed to enhance the rates of treatment compliance and follow-up. Even the existing surveys addressing patient attitudes to dental care at government hospitals in India have been performed mainly among southern and western states of India and their results might not be applicable to the specific sociocultural and economic environment at the state of Bihar [12]. Additionally, the majority of available investigations have concentrated on patient satisfaction as the main outcome variable without fully evaluating knowledge and behavioral aspects that support and determine the level of satisfaction [13]. Having all the three KAB elements integrated in one study gives a more comprehensive picture of the patient experience and creates more actionable information to enhance the services. Moreover, limited literature has been conducted to determine how the knowledge of patients regarding certain forms of dental treatment (like: root canal treatment, prosthetic rehabilitation, scaling and polishing and surgical extractions) can affect their readiness to accept the suggested treatments and adherence to the treatment regimen [14]. Therefore, it is of interest to describe the knowledge, attitude and behavior of patients toward dental treatment at the outpatient department of Government Medical College and Hospital, Patna, Bihar.

## Materials and Methods:

## Study design and setting:

This cross-sectional descriptive study was conducted at the outpatient department (OPD) of the Department of Dentistry, Government Medical College and Hospital (GMCH), Patna, Bihar, India, over a period of Seven months from September 2025 to March 2026. GMCH Patna is a premier government teaching hospital in Bihar, providing multispecialty dental services including oral medicine and radiology, conservative dentistry and endodontics, prosthodontics, periodontics, oral and maxillofacial surgery, orthodontics and Pedodontics. The dental OPD receives an average of 80 to 120 patients daily from urban Patna and surrounding districts. Written informed consent was obtained from all participants in Hindi after comprehensive explanation of the study objectives and assurance of confidentiality.

## Sample size calculation:

Sample size was determined using the formula n = Z^2^pq/d^2^, where Z = 1.96 (95% confidence level), p = estimated proportion of patients with adequate treatment knowledge (assumed 35% based on existing literature), q = 1 - p = 0.65 and d = margin of error (4.5%). The minimum calculated sample size was 456. To accommodate potential incomplete responses, 480 patients were initially approached. Of these, 450 provided complete, analysable responses and constituted the final study sample.

## Participant selection:

Patients attending the dental OPD during the study period were recruited through consecutive sampling across all working days to capture the diversity of patient presentations.

## Inclusion criteria:

[1] Age 18 years and above

[2] Patient attending the dental OPD (new or returning visit)

[3] Willingness to participate and provide written informed consent

[4] Ability to comprehend the questionnaire in Hindi or English

[5] Having received or scheduled to receive at least one dental treatment at the facility

## Exclusion criteria:

[1] Age below 18 years

[2] Patients attending exclusively for radiographic services without clinical consultation

[3] Patients with cognitive or communicative disabilities precluding reliable questionnaire completion

[4] Patients who declined to participate

[5] Hospital staff or dental students seeking treatment at the facility

## Study instrument:

A structured, pretested, interviewer-administered questionnaire was developed specifically for this study through a systematic process of literature review, item generation, expert validation and pilot testing.

## The questionnaire development process involved:

[1] Item generation: Initial items were generated through review of existing validated instruments for dental patient KAB assessment, consultation with senior faculty members in the department and informal patient interviews to identify locally relevant themes and concerns.

[2] Expert validation: The draft questionnaire was reviewed by a panel of six experts (two oral medicine specialists, one prosthodontist, one endodontist, one public health dentist and one psychometrician). Each item was rated for relevance, clarity and cultural appropriateness using a four-point scale. Items achieving a content validity index below 0.80 were revised or removed. The overall scale-level content validity index was 0.88.

[3] Translation: The questionnaire was developed in English, translated into Hindi by two independent bilingual translators and back-translated by a third translator to verify semantic equivalence.

[4] Pilot testing: The instrument was pilot tested on 45 patients (excluded from the main study) to assess comprehension, response patterns and internal consistency. The pilot yielded a Cronbach's alpha of 0.84 for the overall instrument. Average administration time was 15 to 18 minutes.

## The final questionnaire comprised 48 items organized into four sections:

[1] Section A - Socio-demographic Information (10 items): Age, sex, educational qualification, occupation, monthly family income, residential background (urban/semi-urban/rural), marital status, distance from the hospital, mode of transportation and referral source.

[2] Section B - Knowledge About Dental Treatments (14 items): Items assessing knowledge regarding common dental procedures (extraction, filling, root canal treatment, scaling, dentures, crowns, orthodontic treatment), understanding of treatment indications, awareness of treatment alternatives, knowledge of treatment duration and costs at government facilities and understanding of post-treatment care. Each correct response scored one point (maximum 14). Scores categorized as: adequate (≥10), moderate (6-9) and poor (≤ 5).

[3] Section C - Attitude Toward Dental Treatment (14 items): Items assessed on a five-point Likert scale (strongly agree = 5 to strongly disagree = 1) covering dimensions of dental anxiety and fear, trust in dental professionals, perceived quality of care at government facilities, willingness to accept recommended treatments, attitudes toward preventive versus curative care and perceptions of communication and patient-provider interaction. Total attitude score ranged from 14 to 70, categorized as: positive attitude (≥51), neutral attitude (37-50) and negative attitude (≤ 36).

[4] Section D - Behavior Toward Dental Treatment (10 items): Items assessing treatment-seeking triggers (pain/routine/referral), treatment history at the facility, completion of recommended treatments, follow-up compliance, adherence to post-treatment instructions, reasons for non-compliance or treatment abandonment and willingness to recommend the facility to others.

## Data collection procedure:

Data collection was conducted by two trained research assistants who were calibrated through a standardized training protocol. The questionnaire was administered in a face-to-face interview format in a private area within the OPD waiting room to ensure confidentiality and minimize response bias. Patients were approached after registration but before or after their clinical consultation, depending on availability. Care was taken to explain each question clearly without leading the respondent toward any particular answer. Each interview lasted approximately 15 to 20 minutes.

## Statistical analysis:

Data were entered into Microsoft Excel and analyzed using SPSS version 26.0 (IBM Corp., Armonk, NY, USA). Descriptive statistics were expressed as frequencies, percentages and mean ± standard deviation. Chi-square tests were used for associations between categorical variables. One-way ANOVA with Tukey's post-hoc test was used for comparing mean scores across multiple groups. Independent samples t-tests were used for two-group comparisons. Pearson's correlation coefficient was used to assess relationships between continuous knowledge, attitude and behavior scores. Multiple linear regression analysis was performed to identify independent predictors of attitude and behavior scores. Statistical significance was established at p <0.05.waiting periods on patient perception and satisfaction toward dental care services. Assessment of treatment-related knowledge revealed varying levels of awareness among participants. Awareness that decayed teeth could be preserved through restorative fillings was reported by 58.2% of participants. However, awareness regarding root canal treatment as a tooth-saving alternative was considerably lower, observed in only 32.4% of participants. Similarly, only 26.7% of participants were aware that professional scaling procedures do not weaken teeth, reflecting the persistence of common misconceptions regarding periodontal therapy. Knowledge regarding rehabilitation options for missing teeth was relatively better, with 64.2% of participants aware of denture treatment options. Awareness regarding the availability of free or subsidized dental treatment services at GMCH was high and reported by 71.8% of participants. In contrast, only 28.4% of participants were aware of the presence of multiple dental specialties available at GMCH. Knowledge of correct post-extraction care instructions was also inadequate, with only 38.9% of participants demonstrating appropriate awareness. Overall, the findings indicate moderate awareness regarding basic dental treatment procedures but poor understanding of specialized dental care services and preventive treatment concepts among the study population.

## Results:

A total of 450 patients completed the study. The socio-demographic profile and treatment knowledge levels are presented in Table 1 while the attitude scores and item-level responses are summarized in Table 2 Behavioral aspects related to dental treatment seeking, treatment compliance and follow-up patterns are detailed in Table 3 Among the returning patients (n = 202), those who reported receiving adequate explanations regarding their dental treatment from the treating dentist demonstrated significantly higher compliance rates (52.3%) compared to those who did not receive adequate explanations (29.4%, p < 0.001). Furthermore, patients who experienced longer waiting times before consultation exhibited significantly poorer attitude scores. Patients waiting for more than two hours had a mean attitude score of 44.18 ± 10.42, whereas those waiting for less than one hour had significantly higher scores of 52.86 ± 8.64 (p <0.001), indicating the negative impact of prolonged waiting periods on patient perception and satisfaction toward dental care services. Assessment of treatment-related knowledge revealed varying levels of awareness among participants. Awareness that decayed teeth could be preserved through restorative fillings was reported by 58.2% of participants. However, awareness regarding root canal treatment as a tooth-saving alternative was considerably lower, observed in only 32.4% of participants. Similarly, only 26.7% of participants were aware that professional scaling procedures do not weaken teeth, reflecting the persistence of common misconceptions regarding periodontal therapy. Knowledge regarding rehabilitation options for missing teeth was relatively better, with 64.2% of participants aware of denture treatment options. Awareness regarding the availability of free or subsidized dental treatment services at GMCH was high and reported by 71.8% of participants. In contrast, only 28.4% of participants were aware of the presence of multiple dental specialties available at GMCH. Knowledge of correct post-extraction care instructions was also inadequate, with only 38.9% of participants demonstrating appropriate awareness. Overall, the findings indicate moderate awareness regarding basic dental treatment procedures but poor understanding of specialized dental care services and preventive treatment concepts among the study population.

## Discussion:

The current research has offered a detailed evaluation of knowledge, attitude and behavior of patients on dental treatment in one of the largest government dental hospitals in Bihar and identified some of the most critical areas that need to be intervened upon in order to enhance the use of dental treatment, treatment completion and patient outcomes. The results indicate that the population has large gaps in knowledge, moderately favourable but weak attitudes and largely reactive treatment-seeking behaviour and poor follow-up compliance. The fact that only 34.2 percent of the respondents showed proper knowledge about dental treatments is in line with research conducted by other government healthcare institutions in India whereby the treatment knowledge levels of 25 to 40 percent among dental outpatients have been documented. The very low knowledge of root canal treatment (32.4) and the prevalence of a false belief about scaling causing weakening of teeth (73.3% false) are the particular knowledge gaps with direct clinical implications [15]. Unconscious patients who do not know that root canal procedure will help save their teeth will default to root canal extracted teeth that could be treated which will cause unnecessary loss of teeth and consequent prosthetic rehabilitation which will end up consuming the already limited resources at governmental facilities. On the same note, the myth regarding scaling might make the patients reluctant to accept prescribed periodontal therapy, which is permitting diseases to develop [16]. The prevalence of dental anxiety (62.4 percent) is also in line with cases of anxiety prevalence in the population with little or no dental contacts and environments where the dental experience has been largely attributed to emergency pain management issues and not to any positive experience in preventive care. The dental anxiety within such groups is more likely to be sustained in a vicious cycle: anxiety makes people avoid regular dental treatment, which causes the disease to develop and only shows it on the instances of acute pain, which in turn entails more invasive, uncomfortable treatment that causes more anxiety [17]. To eliminate this cycle it is necessary to have systematic application of anxiety management techniques such as better communication with patients, transparency in procedures and establishment of supportive clinical environments in the government dental departments. The attitude expressed by 57.8% of the participants about the need to seek dental care only when in pain being experienced is perhaps the most basic attitudinal barrier to effective oral healthcare among this group of people. This is a paradigm of seeking dental care as a result of painful experiences and it is firmly ingrained in the healthcare culture of most developing areas and strengthened by the opportunity costs of seeking dental care in terms of travel time, lost wages and lengthy waits in government centers, which the patient is not ready to spend time on without acute symptoms [18]. The change of such a paradigm needs a long-term community-based health education on the advantages and cost-effectiveness of preventative and early interventive dental care. The attitude that governmental dental services are poorer than those offered by the private sector as seen by 36.2% of the sample population is a troubling fact that might push people to abandon the use of government services and seek options that might be unregulated by the government. Such an impression can be stoked by increased wait times, the perceived scarcity of resources, or benchmarked against a physical infrastructure and facilities of the private dental practices [19]. Nonetheless, the conclusion that 64.9 percent would recommend the facility to others indicates that the firsthand experience of the level of clinical care mitigates the impact of negative preconceptions to a certain extent, which emphasizes the role of positive patient experiences as a source of institutional trust. The poor treatment compliance of 38.7 and the poor attendance of follow-up of 20.9 are the essential issues of under-treatment at this facility. The unfinished treatment of the teeth does not only fail to reach the desired therapeutic results but, in fact, can result in the further deterioration of the condition of the patient: an uncompleted root canal, a half-finished prosthetic rehabilitation, or a half-scaled dentition all can lead to a worse condition than its state before the treatment [20]. The identification of financial constraints (30.4%), length of waiting period (26.1%) and relief of the symptoms following first treatment (20.3%) as the core reasons of non-compliance gives a viable object of intervention. The high interrelation between the scores of knowledge and attitude (r = 0.52) and between the scores of knowledge and attitude and the treatment compliance are the results of the interdependence between knowledge and attitude scores. Education and knowledge also act as the determinants that operate on attitudes and subsequently affect the behavioral choices [21]. The fact that the multiple regression model identified education level and knowledge scores as the most significant independent predictors of the scores in attitude validates the importance of patient education as one of the methods of enhancing the overall engagement in dental care. The fact that the independent contribution of prior positive dental experience is positive also indicates that quality improvement efforts aimed at improving the experience of the patient during their first visit have a cascading positive effect on future attitudes and behaviors [22]. The strong proportion of self- medication (59.6) and consultation with traditional healers (18.2) followed by a dental professional demonstrates the current healthcare- seeking pecking order in the target population, according to which dental professionals are approached only when other non-professional healthcare providers are unable to help. This lateness in presentation is part of the later stages of the disease where the patients eventually report to the dental OPD and consequently have to undergo more complicated, time consuming and resource demanding treatments that would have otherwise not been necessary at the earlier stages [23]. The 52.0% patients who took over one month to seek dental treatment after the onset of symptoms depict a huge period of preventable disease development. The fact that when patients are given proper treatment explanations, they showed a considerable high rate of compliance proves the vital importance of communication between patients and doctors in the context of treatment adherence. Effective communication about the diagnosis, the rationale behind the treatment, the anticipated results, the steps involved in the procedure and the post-operative instructions will enable the patients to make informed decisions and create realistic expectations which will reduce the anxiety and encourage them to attend the follow-up [24]. Structured communication guidelines and additional learning resources would ensure patient knowledge without the need to spend much more time in the consultation room in the setting of a high-volume government dental OPD, where time is constrained and providers might not have enough time to establish communication with patients. There are a number of constraints of this research that should be taken into consideration. The use of cross-sectional design does not allow making a causal inference about the relationship between knowledge, attitude and behavior. The sampling method used by the facility is restricted to the generalizability with the rest of the population that might not utilize dental services at all. Self-reported compliance data and behavioral data could suffer social desirability bias and recall bias. Although the interviewer-administered format is required because of limited literacy levels of the participants, it can expose the interviewer bias even after undergoing standardized training [25]. Longitudinal research that keeps following the factual completion rates of treatments associated with educational interventions would be more informative in intervention design.

## Conclusion:

We show that patients attending the dental OPD at Government Medical College and Hospital, Patna exhibit inadequate knowledge of dental treatments despite relatively positive attitudes, with care-seeking behavior largely reactive and pain-driven. Poor treatment compliance and follow-up adherence further compromise therapeutic outcomes, highlighting significant gaps in effective healthcare utilization. Targeted patient education, improved dentist-patient communication and structured preventive care strategies are essential to enhance treatment acceptance and overall oral health outcomes.

## Figures and Tables

**Table 1:** Socio-demographic Characteristics and Dental Treatment Knowledge Levels (n = 450)

**Characteristic**	**n (%)**	**Adequate Knowledge n (%)**	**p-value**
**Age Group**			
18-25 years	118 (26.2%)	52 (44.1%)	
26-40 years	164 (36.4%)	62 (37.8%)	0.008
41-55 years	108 (24.0%)	28 (25.9%)	
> 55 years	60 (13.3%)	12 (20.0%)	
**Sex**			
Male	256 (56.9%)	82 (32.0%)	0.182
Female	194 (43.1%)	72 (37.1%)	
**Education Level**			
Illiterate	68 (15.1%)	6 (8.8%)	
Primary school	82 (18.2%)	14 (17.1%)	
Secondary school	108 (24.0%)	34 (31.5%)	<0.001
Higher secondary	96 (21.3%)	44 (45.8%)	
Graduate and above	96 (21.3%)	56 (58.3%)	
**Residential Background**			
Urban	168 (37.3%)	78 (46.4%)	
Semi-urban	138 (30.7%)	46 (33.3%)	< 0.001
Rural	144 (32.0%)	30 (20.8%)	
**Monthly Family Income**			
< ₹10,000	152 (33.8%)	28 (18.4%)	
₹10,000-25,000	172 (38.2%)	64 (37.2%)	< 0.001
> ₹25,000	126 (28.0%)	62 (49.2%)	
**Distance from Hospital**			
< 10 km	148 (32.9%)	62 (41.9%)	
10-50 km	168 (37.3%)	58 (34.5%)	0.033
> 50 km	134 (29.8%)	34 (25.4%)	
**Overall Knowledge Level**			
Adequate (≥10)	154 (34.2%)	-	-
Moderate (6-9)	182 (40.4%)	-	-
Poor ( ≤ 5)	114 (25.3%)	-	-
Knowledge Score, Mean ± SD	7.16 ± 3.12	-	-

**Table 2:** Attitude toward dental treatment at GMCH dental OPD (n = 450)

**Panel A: Attitude Category Distribution**	**n (%)**
Positive attitude ( ≥ 51)	236 (52.4%)
Neutral attitude (37-50)	148 (32.9%)
Negative attitude (≤ 36)	66 (14.7%)
Attitude Score, Mean ± SD	48.72 ± 9.86
Panel B: Item-Level Responses (% Agree/Strongly Agree)	
I feel anxious/fearful about dental treatment	62.40%
I trust the dental professionals at this hospital	68.20%
I believe extraction is the best solution for toothache	41.30%
**I think dental treatment at government hospitals is inferior to private**	36.20%
I am satisfied with the explanation provided before treatment	54.70%
I feel comfortable asking questions to the dentist	42.20%
I believe preventive dental care is important	56.40%
**I would accept root canal treatment over extraction if recommended**	48.90%
I am satisfied with the waiting time at the dental OPD	32.70%
I believe dental treatment is necessary only when there is pain	57.80%
I feel the facility has adequate equipment and materials	46.20%
I would recommend this facility to family/friends	64.90%
I feel embarrassed about the condition of my teeth	38.40%
I believe regular dental check-ups are worthwhile	52.00%
Panel C: Attitude Score by Subgroup	Mean ± SD
Education - Illiterate	41.24 ± 8.92
Education - Graduate and above	54.38 ± 8.74
Residence - Urban	51.86 ± 9.12
Residence - Rural	44.58 ± 10.24
Previous dental visit - Yes	51.42 ± 8.76
Previous dental visit - No (First visit)	44.28 ± 10.54

**Table 3:** Treatment-seeking behavior, compliance and associated factors (n = 450)

**Panel A: Treatment-Seeking Behavior**	n (%)
**Primary Reason for Current Visit**	
Pain/emergency	284 (63.1%)
Specific complaint (swelling/bleeding/mobility)	78 (17.3%)
Routine check-up/preventive care	36 (8.0%)
Referral from other department/physician	32 (7.1%)
Continuation of ongoing treatment	20 (4.4%)
**Visit History**	
First-ever dental visit in life	106 (23.6%)
First visit to this facility	142 (31.6%)
Returning patient at this facility	202 (44.9%)
Self-Medication Before Visiting Dentist	268 (59.6%)
Consulted Traditional/Alternative Healer First	82 (18.2%)
Delay > 1 Month Between Symptom Onset and Visit	234 (52.0%)
**Panel B: Treatment Compliance**	
Completed all recommended treatment	174 (38.7%)
Partially completed treatment	152 (33.8%)
Did not return after first visit	124 (27.6%)
**Reasons for Non-Compliance (among non-compliant, n = 276)**	
Financial constraints	84 (30.4%)
Long waiting time/multiple visits required	72 (26.1%)
Symptom relief after initial treatment	56 (20.3%)
Fear of further procedures	38 (13.8%)
Distance/transportation difficulty	46 (16.7%)
Work/family obligations	34 (12.3%)
Dissatisfaction with initial treatment experience	22 (8.0%)
**Follow-Up Appointment Adherence**	
Always attends scheduled follow-up	94 (20.9%)
Sometimes attends follow-up	128 (28.4%)
Rarely/never attends follow-up	228 (50.7%)
Panel C: Correlation Analysis (Pearson's r)	
Knowledge score vs. Attitude score	r = 0.52, p < 0.001
Knowledge score vs. Compliance (complete = 1)	r = 0.41, p < 0.001
Attitude score vs. Compliance (complete = 1)	r = 0.46, p < 0.001
**Panel D: Multiple Regression - Dependent Variable: Attitude Score**	**β**
Education level	0.32
Knowledge score	0.28
Previous dental experience (positive = 1)	0.18
Urban residence	0.14
Monthly income	0.11
Age	-0.06
Sex	0.04
R^2^ = 0.48, F = 58.34, p < 0.001	
